# Femtosecond Mode-locked Fiber Laser at 1 μm Via Optical Microfiber Dispersion Management

**DOI:** 10.1038/s41598-018-23027-9

**Published:** 2018-03-16

**Authors:** Lizhen Wang, Peizhen Xu, Yuhang Li, Jize Han, Xin Guo, Yudong Cui, Xueming Liu, Limin Tong

**Affiliations:** 1 State Key Laboratory of Precision Measurement Technology and Instruments, Tsinghua University, Beijing, 100084 China; 2 State Key Laboratory of Modern Optical Instrumentation, College of Optical Science and Engineering, Zhejiang University, Hangzhou, 310027 China; 3 Department of Physics, Tsinghua University, Beijing, 100084 China

## Abstract

Mode-locked Yb-doped fiber lasers around 1 μm are attractive for high power applications and low noise pulse train generation. Mode-locked fiber lasers working in soliton and stretched-pulse regime outperform others in terms of the laser noise characteristics, mechanical stability and easy maintenance. However, conventional optical fibers always show a normal group velocity dispersion around 1 μm, leading to the inconvenience for necessary dispersion management. Here we show that optical microfibers having a large anomalous dispersion around 1 μm can be integrated into mode-locked Yb-doped fiber lasers with ultralow insertion loss down to −0.06 dB, enabling convenient dispersion management of the laser cavity. Besides, optical microfibers could also be adopted to spectrally broaden and to dechirp the ultrashort pulses outside the laser cavity, giving rise to a pulse duration of about 110 fs. We believe that this demonstration may facilitate all-fiber format high-performance ultrashort pulse generation at 1 μm and may find applications in precision measurements, large-scale facility synchronization and evanescent-field-based optical sensing.

## Introduction

Ultrafast lasers have opened up many important applications^1^. Mode-locked Yb-doped fiber lasers in the net-normal and all-normal dispersion regimes, named as similariton and dissipative soliton lasers, are very attractive for high power applications such as material processing and sources for visible/UV light generation^2,3^, due to the large pulse energy, high environmental robustness and the compactness, particularly for the all-PM-fiber lasers developed recent years^4–6^. Meanwhile, for applications requiring low noise pulse trains such as optical frequency combs and ultralow timing jitter clock signal generation, soliton and stretched-pulse fiber lasers outperform others in terms of the noise characteristics^7^. Especially, the stretched-pulse ultrafast lasers alleviate the excessive accumulated nonlinear phase shift, and thus can overcome the barrier of the soliton area-theorem associated with soliton lasers, due to the considerable breathing ratio both in temporal and spectral domain. Usually, these two kinds of lasers require a negative or near zero net cavity dispersion.

However, conventional optical fibers, both Yb doped and undoped single-mode fibers (SMF), always show a normal second-order dispersion (*β*_2_) around 1 μm, because of the large normal material dispersion and the relatively weak waveguide dispersion. Typical approaches to circumvent this difficulty are to adopt free-space components such as grating/prism pairs^8–10^. Photonic crystal fibers and tapers drawn from them exhibit highly flexible dispersion by modifying the geometric design, and are compatible with fiber components. They have been proposed and demonstrated in ultrashort pulse generation and propagation, ranging from ultrashort pulse generation, soliton compression, waveform shaping to supercontinuum generation^11–16^. However, the insertion loss is relatively high so far.

Benefitting from their wavelength-scale dimensions and high index contrast, optical microfibers drawn from conventional optical fibers exhibit the advantages of tight optical confinement, and thus a high effective optical nonlinearity and a strong diameter-dependent waveguide dispersion. Also, these microfibers can offer relatively strong and easily accessible evanescent field, easy fabrication, and excellent compatibility with conventional fiber systems^17,18^. Particularly, the strong diameter-dependent dispersion (i.e., waveguide dispersion) can be used for counterbalancing the material dispersion around 1 μm, giving rise to a negative *β*_2_, which is highly desired for dispersion management required for soliton and stretched-pulse operation of mode-locked Yb-doped fiber lasers. This delicate dispersion management can not only overcome the gain bandwidth limitation for the ultrashort pulse generation^19^, but also enable a relatively low intensity noise and an ultralow timing-jitter operation, since the Gordon-Haus jitter can dominate in ultrafast fiber lasers, which directly relies on the net cavity dispersion^7,20,21^.

Ultrafast lasers have been built with optical microfibers combined with fast saturable absorbers, in which the microfibers were employed as a support for the saturable absorber, rather than a dispersion management component^22,23^. Optical microfibers have been proposed for dispersion compensation, and have been demonstrated in lasers with a pulse duration of a few picoseconds^24,25^. Now we show in the present work that a short piece of optical microfibers could be conveniently incorporated both inside and outside of the ultrafast Yb fiber laser cavity with ultralow insertion loss, serving multifunctionally as a dispersion management, spectrally broadening and dechirping component, and thus enabling a pulse duration of about 110 fs. Benefitting from the diameter-dependent dispersion, ultralow insertion loss and easy fabrication of optical microfibers, we believe this work could provide a convenient and versatile scheme for dispersion management in ultrafast fiber lasers. This scheme is fully compatible with conventional fiber components and can be widely adopted in high-performance ultrashort pulse generation and diverse ultrashort pulse-based applications.

## Results

### Optical microfiber loss and dispersion characterization

**Figure 1:**
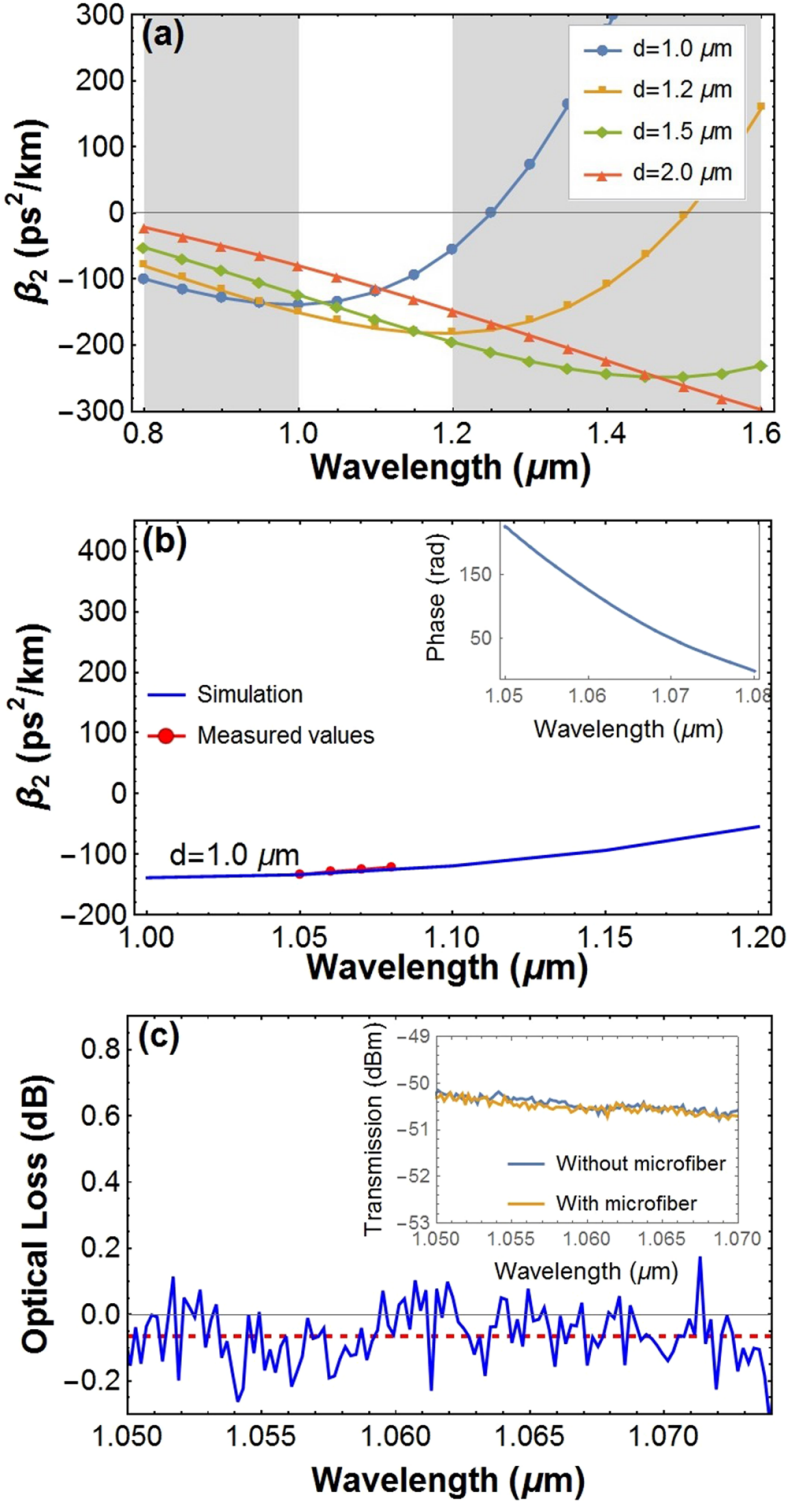
(**a**) Calculated *β*_2_ for optical microfibers with a diameter of 1.0, 1.2, 1.5, and 2.0 μm, respectively. (**b**) The measured (red line) and the calculated (blue line) *β*_*2*_ for an optical microfiber with a diameter of 1.0 μm, which was chosen in accordance with the one used in the experiment shown in Fig. 2. The inset shows the measured relative phase delay, from which we obtained the *β*_*2*_. (**c**) The measured insertion loss of the optical microfiber in (b). The inset shows the transmission spectra of fibers spliced with and without optical microfibers, and the difference of the two curves is the insertion loss. The red dashed line gives the average loss, i.e., −0.06 dB. The points above the zero-loss line are attributed to the spectral fluctuations of the broadband source we used.

The calculated *β*_2_ of optical microfibers^26^ is shown in Fig. 1(a). It can be seen that the *β*_2_ is always negative around the wavelength of 1060 nm, ranging from −160 ps^2^/km to −60 ps^2^/km, for optical microfibers with a diameter between 1 μm and 2 μm. This simulation results imply that the optical microfibers would be efficient for dispersion management, since the *β*_2_ can be several times larger than that of conventional SMFs (e.g., HI1060 and HI1060FLEX, Corning), which are always positive (claimed to be 23 and 30 ps^2^/km at 1060 nm, respectively, in the datasheet). This feature of diameter-dependent dispersion, which can be designed and controlled readily during the drawing process, is the first advantage of optical microfibers for dispersion management.

For the as-fabricated 25 cm-long optical microfiber with a diameter of 1.0 μm, the group delay dispersion (GDD) was measured to be −0.030 ps^2^ and the *β*_2_ of about −120 ps^2^/km at 1060 nm. This *β*_2_ value matched closely with the simulation results, shown in Fig. 1(b). The measurement results indicated that this optical microfiber was able to counterbalance the normal dispersion of a 1.6 m-long single-mode fibers (HI1060, Corning).

Dispersion management components with a low insertion loss would be beneficial for many applications. Especially, it can be helpful for a high intracavity pulse power, and thus a low relative intensity noise and low timing jitter operation of mode-locked lasers^27,28^. Benefitting from the excellent surface quality during the drawing process and the continuously connected conventional fibers via adiabatic tapering regions, optical microfibers could show a very low intrinsic loss and high quality splicing with other fiber pigtails. Figure 1(c) shows the measured insertion loss, including the intrinsic and splicing loss, with an average value of −0.06 dB (i.e., a transmission of 98.5%) over the full spectral range. Note that this ultralow insertion loss can hardly be achieved with other dispersion compensation components. This feature of ultralow insertion loss is the second advantage of optical microfibers for dispersion management.

### Femtosecond fiber laser building and characterization

**Figure 2:**
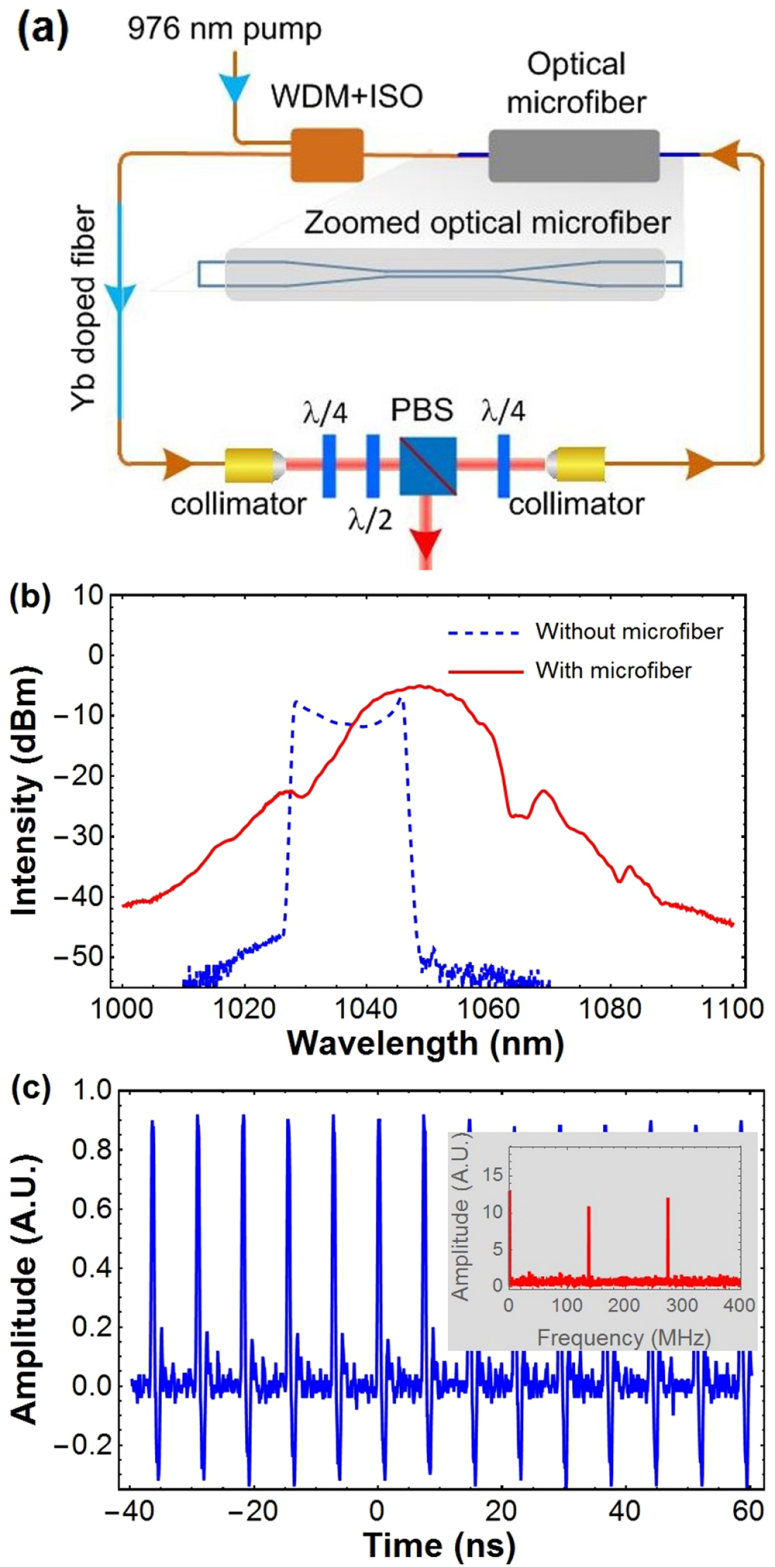
(**a**) Schematic for an ultrafast fiber laser incorporating an optical microfiber for dispersion management. The pump at 976 nm entered the laser cavity via a 980/1060 nm WDM/Isolator hybrid and pumped a 15 cm-long Yb doped fiber (Yb1200, Liekki). The waveplates and the PBS between the two collimators worked as an artificial fast saturable absorber for the mode-locked laser operation via nonlinear polarization evolution. A sealed optical microfiber was spliced with negligible optical losses for cavity dispersion management. The zoomed-in figure shows the details of the sealed optical microfiber. (**b**) Typical output optical spectrum at a pump power of about 150 mW for the laser cavity incorporated with a 25 cm long optical microfiber with a diameter of about 1 μm. Also showed the dashed blue line standing for the laser without the optical microfiber, for a comparison. (**c**) The waveform from a 1 GHz photodetector displayed on a 6 GHz-bandwidth oscilloscope. The Fourier-transform spectrum shows as the inset, with a fundamental frequency of 120 MHz, corresponding to a cavity length of 1.7 m.

We built ultrafast lasers incorporating the optical microfibers for dispersion management at 1 μm. The laser setup is shown in Fig. 2(a). A piece of 15 cm-long Yb-doped fiber (Yb1200, Liekki) served as the gain medium, and a 980/1060 nm WDM/Isolator hybrid was used for efficient pump light coupling and a unidirectional operation of the ultrafast laser. The waveplates and the PBS between the two collimators worked as the artificial fast saturable absorber by using nonlinear polarization evolution. The optical microfiber measured in Fig. 1(b) was incorporated into the laser cavity between the collimator 2 and WDM/Isolator hybrid with a negligible splicing loss. All of the free-space components can be conveniently replaced by the all-fiber format counterparts, but at present we would like to employ the free-space ones for their precise polarization manipulation capability. The output ultrashort pulses were taken behind the rejection port of PBS.

The pigtails of the WDM/Isolator hybrid and the collimators were HI1060, and the *β*_2_ was measured to be 22 ps^2^/km, closely agreeing with the datasheet. The total length of the cavity was measured to be 1.7 m, and the net cavity dispersion was calculated to be −0.006 ps^2^.

Mode-locked laser operation could be initiated by manipulating the three waveplates at a pump power of about 600 mW, however, in the multipulse regime. The mode-locked state could persist even when the pump power was gradually reduced to 150 mW, and reached single-pulse operation. The optical spectrum of the output pulses, shown as the red line Fig. 2(b), was in great contrast to the rectangular spectrum without the optical microfiber inside the laser cavity (blued dashed), which was a typical spectrum for all-normal dispersion operation. The output pulses were detected by a fast photodetector (Model: DET01CFC/M, Thorlabs) and displayed on a 6 GHz oscilloscope (Model: DPO 70604, Tektronics) to monitor the temporal waveforms of the ultrashort pulse trains. It can be seen in Fig. 2(c) that well periodic signal was observed and the Fourier transform frequency spectrum showed a repetition rate of 120 MHz. It should be noted that the spectrum shown in Fig. 2(b) is more soliton-like, rather than stretched-pulse like. This can be explained by the spectral breathing of the pulse along the fiber, which is very common in dispersion-managed ultrafast lasers^29^.

### Dechirping outside the cavity via optical microfiber

**Figure 3:**
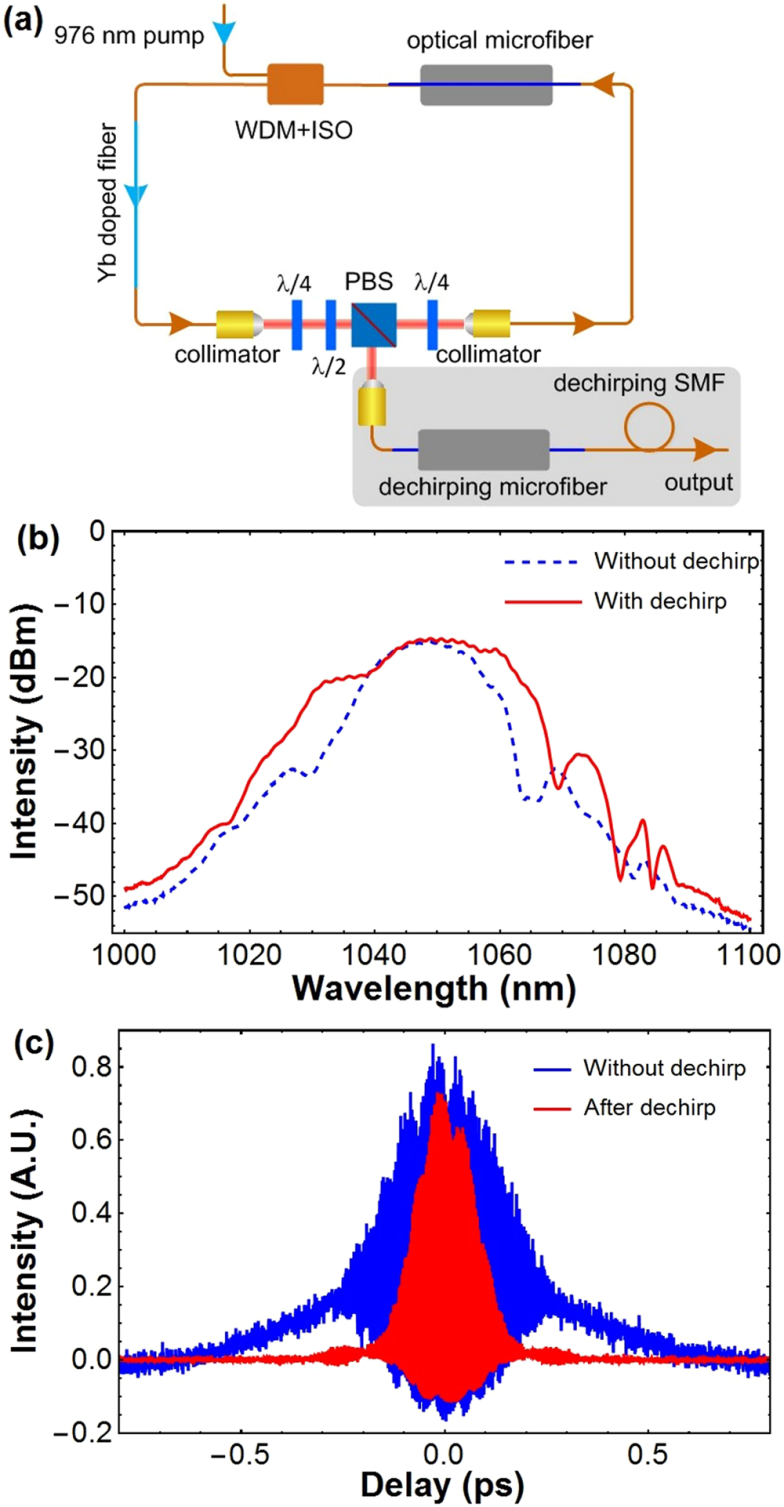
(**a**) Schematic for an ultrafast fiber laser with a dechirping microfiber and SMF at the output pigtail. (**b**) The output optical spectra for lasers with (red solid) and without (blue dashed) the dechirping optical microfiber. The pump power was about 150 mW, and the output power was measured to be 60 mW. (**c**) The interferometric autocorrelations for lasers with (red) and without (blue) the dechirping optical microfiber. The dechirping optical microfiber had identical parameters with the one inside the cavity shown in Fig. 2(a), and the dechirping SMF was a piece of about 30 cm long HI1060 (Corning).

It is worth to note that optical microfibers can also be adopted outside the laser cavity to broaden the output spectrum and to dechirp the pulses simultaneously. It is because the optical microfibers could also display a large Kerr nonlinearity due to the μm-scale diameter and thus a high optical confinement. This feature could be very beneficial for pulse compression. To further demonstrate this capability and to characterize the pulse duration of the laser, we adopted another optical microfiber with the same parameters as the one in the cavity, spliced with a piece of following SMF in the dechirping arm after the rejection port of PBS as shown in Fig. 3(a). The length of SMF was cut short gradually to obtain the shortest pulse according to the autocorrelation trace. Figure 3(b) and (c) showed the optical spectra and the interferometric autocorrelations (IACs), respectively, for with and without dechirping optical microfiber for a comparison. It can be seen in Fig. 3(b) that the optical spectrum was broadened apparently, from 15 nm to 20 nm, for the case with the dechirping optical microfiber due to the nonlinear interactions along the microfiber. Meanwhile, optical microfiber also greatly reduced the chirp of the pulse, and the shortest pulse duration can be inferred from the IAC shown in Fig. 3(c) was about 110 fs. This dual role of spectrally broadening and pulse dechirping is the third advantage of optical microfibers for dispersion management, and could be very useful for ultrashort pulse generation.

## Discussion

In conclusion, we showed that optical microfibers with a diameter-dependent dispersion could be adopted for dispersion management required for ultrafast lasers. This scheme was shown in femtosecond mode-locked Yb-doped fiber lasers around 1 μm incorporated optical microfibers both inside the laser cavity and in the dechirping arm. Features such as the diameter-dependent dispersion, ultralow insertion loss, and high Kerr nonlinearity, easy fabrication, and fully compatible splicing with conventional fiber components promise that pigtailed optical microfibers could be widely used in ultrafast fiber lasers, especially in all-fiber format version. Optical microfibers can also be functionalized with fast saturable absorbers, including carbon nanotubes, graphene and many others^30–32^, serving both as a dispersion management component and a saturable absorber, and thus enable an all-fiber format high performance turn-key laser system for ultrashort pulse generation and applications in precision measurements and facility synchronization. Beside, due to the easily accessible evanescent field around the optical microfibers, this kind of mode-locked fiber lasers can also find applications in evanescent-based optical sensing via ultrashort pulses^33^.

## Methods

The optical microfibers were drawn from conventional SMF (HI1060, Corning) via the traveling-stage taper-drawing scheme as described elsewhere^34,35^. The optical microfiber waist with a uniform diameter of about 1.0 μm and a length of 25 cm was continuously connected with SMF pigtails via adiabatic tapering regions with a length of about 8 cm. The as-fabricated microfiber as well as the tapering regions were kept straight and sealed inside a plastic box to prevent possible damage and air pollution.

We measured the group delay dispersion (GDD) of the as-fabricated optical microfibers using the white-light interferometry^36,37^. The relative phase delay was obtained according to the recorded interference spectrum and amplified spontaneous emission (ASE) background by a Fourier transform-based phase retrieval technique, shown in the inset of Fig. 1(b), and the GDD and *β*_2_ can be inferred from this relative phase delay.

### Data availability

The authors declare that all the data in this manuscript are available.
